# Maximal pulmonary ventilation and lactate affect the anaerobic performance in young women exposed to hypobaric hypoxia

**DOI:** 10.3389/fphys.2023.1110477

**Published:** 2023-02-08

**Authors:** Valeria Páez, Maria Rodriguez-Fernandez, Juan Silva-Urra, Cristian Núñez-Espinosa, Morin Lang

**Affiliations:** ^1^ Center for Research in Physiology and Medicine of Altitude, Biomedical Department, Faculty of Health Sciences, Universidad de Antofagasta, Antofagasta, Chile; ^2^ Schools of Engineering, Medicine and Biological Sciences, Institute for Biological and Medical Engineering, Pontificia Universidad Católica de Chile, Santiago, Chile; ^3^ Network for Extreme Environmental Research (NEXER), Universidad de Antofagasta, Antofagasta, Chile; ^4^ School of Medicine, Universidad de Magallanes, Punta Arenas, Chile; ^5^ Centro Asistencial Docente y de Investigación, CADI-UMAG, Punta Arenas, Chile; ^6^ Department of Rehabilitation Sciences and Human Movement, Faculty of Health Sciences, Universidad de Antofagasta, Antofagasta, Chile

**Keywords:** high altitudes, hypoxia, exercise, multiple-sprint performance, lactate, anaerobic test, pulmonary ventilation

## Abstract

**Background:** Athletes, tourists, and mining workers from all over the world ascend daily to an altitude greater than 3.000 meters above sea level to perform different activities, all of which demand physical effort. A ventilation increase is the first mechanism once the chemoreceptors perceive hypoxia, and is key to maintaining blood oxygen levels during acute exposure to high altitudes and to buffering lactic acidosis during exercise. It has been observed that gender is a variable that can influence the ventilatory response. Still, the available literature is limited due to the few studies considering women as study subjects. The influence of gender on anaerobic performance and its effects under high altitudes (HA) environments have been poorly studied.

**Objective:** The objectives of this study were to evaluate anaerobic performance in young women exposed to high altitudes and to compare the physiological response to multiple sprints between women and men measured by ergospirometry.

**Methodology:** Nine women and nine men (22.9 ± 3.2 years old) carried out the multiple-sprint anaerobic tests under two conditions, sea level and high altitudes.

**Results:** In the first 24 h of exposure to a high altitudes, lactate levels were higher in women than those in men (2.57 ± 0.4 Mmol/L, 2.18 ± 0.3 Mmol/L, respectively; *p* < 0.05). Second, women had a decreased ventilatory response in exposure to high altitudes compared to men (*p* > 0.005). Third, there is a positive correlation between lactate levels prior to an anaerobic test and the ventilatory response developed by subjects at high altitudes (R2 = 0.33, slope = -41.7, and *p* < 0.05). Lastly, this ventilatory response can influence VO_2peak_ (R2 = 0.60, slope = 0.02, and *p* < 0.001).

**Conclusion:** This study provides insights into the mechanisms behind the reduced respiratory capacity observed in women during an anaerobic exercise test at high altitudes. An acute response to HA showed a greater work of breathing and increased the drive ventilatory response. It is possible to postulate the differences in the fatigue-induced metaboreflex of the respiratory muscles and aerobic–anaerobic transition between genders. These results on multiple sprint performance and the influences of gender in hypoxic environments deserve further investigation.

## 1 Introduction

Athletes, tourists, and mining workers from all over the world ascend daily to an altitude greater than 3000 m above sea level (m.a.s.l) to perform different activities, all of which demand physical efforts at diverse intensities. Any physical activity carried out at high altitudes (HA) will generate a greater intensity than the same exercise performed at sea level (SL) due to an imbalance of the organism’s homeostasis induced by hypobaric hypoxia (Mazzeo, 2008; Lang et al., 2021). Hypoxemia and subsequent hypoxia are consequences of lower inspired pressure of oxygen (PiO_2_) caused by the drop in the arterial pressure of oxygen (PaO_2_) (Luks, 2015). An increase in ventilation is the first response mechanism once chemosensitive structures in the medulla and carotid bodies perceive hypoxia (Holwerda and Vianna, 2019). A ventilatory response (VR) is key to maintaining blood oxygen levels during acute exposure to HA and buffering lactic acidosis during exercise.

Multiple mechanisms can influence the VR to hypoxia (Dempsey et al., 2006; Harms, 2007; Moreno et al., 2014; Holwerda and Vianna, 2019; Lam et al., 2019). Applying the circulatory occlusion method that induces local hypoxia in the locomotor muscles during exercise, researchers have revealed that the afferent feedback from contracting skeletal muscle to the central respiratory system contributes to exercise hyperpnea (Holwerda and Vianna, 2019; Lam et al., 2019). In addition, metaboreflex can also be activated by fatigue of the respiratory muscles during high-intensity exercise (>80% maximal oxygen consumption, VO_2max_), allowing the blood flow to be redistributed, favoring the perfusion of the respiratory muscles, and decreasing the perfusion of the locomotor muscles, enhancing the oxygenation of hypoxemic blood (Dempsey et al., 2006; Harms, 2007). This phenomenon is also observed in subjects in continuous hypoxia due to cardiovascular and respiratory diseases, such as heart failure and chronic obstructive pulmonary disease (Dempsey et al., 2006; Moreno et al., 2014). At HA, this mechanism could help maintain respiratory muscles’ oxygenation, delaying respiratory fatigue and favoring a higher respiratory frequency, and postponing respiratory acidosis during high-intensity exercises.

Many team and individual sports require the development of aerobic and anaerobic capacities for optimal performance, which broadens the connection of altitude/hypoxia with training and measures of aerobic capacity, incorporating the anaerobic component as part of the physical condition of an athlete (Girard et al., 2017). Alterations in an anaerobic exercise in multiple sprints at HA, such as the repetition of maximal effort above 3000 m.a.s.l, where the inspired fraction of oxygen (FiO_2_) is reduced by 13.3%, have been observed (Girard et al., 2017). Cycling studies have shown a decreased ability to maintain the power output toward the end of each exercise sprint at HA compared to a normoxic condition or SL, and other studies revealed reductions in maximal or mean power output compared to normoxia during repeated sprint exercises (6–10 x 5–10 s cycling sprints—20–30 s recovery) (Girard et al., 2017).

On the other hand, it has been observed that gender is another variable that can influence the VR to HA. Women have mechanical respiratory restrictions that can affect their response to a geographically high altitudes and exercise. The literature available is limited due to the few studies considering women as the study subjects. A strong relationship between gender and VR has been reported (Goldberg et al., 2017). Women have smaller lungs and airways, causing expiratory flow limitation (Dominelli et al., 2013). Moreover, women develop exercise-induced arterial hypoxemia during a constant load test (∼85% VO_2max_) associated with lower ventilatory capacity, but men were not measured in the study (Dominelli et al., 2013).

Hypoxia is perceived by mammalian cells, causing metabolic adaptations to compensate for hypoxemia with or without exercise (Semenza et al., 1996; Smith et al., 2008). An important intracellular adaptation to hypoxia is the transition from oxidative phosphorylation to glycolysis as the primary means of adenosine triphosphate (ATP) generation. The hypoxia-inducible factor 1 (HIF-1) is important since it regulates the expression of a variety of genes involved in glycolysis, such as lactate dehydrogenase A (LDHA), aldolase A (ALDA), and enolase 1 (ENO1) (Semenza et al., 1996; Dominelli et al., 2013). Aerobic glycolysis is a fairly efficient oxidative mechanism for the production of ATP. However, under hypoxic conditions, the preferred mechanism is likely anaerobic glycolysis, which leads to the lower production of ATP and loss of isocapnic buffering, rapidly causing respiratory acidosis and, subsequently, metabolic acidosis. Indirect indicators of these processes are the respiratory exchange ratio (RER) and lactate during exercise.

Hypobaric hypoxia exposure is a physiological challenge for humans. Adequate ventilatory capacity is required to maintain the arterial oxygen pressure during exposure to acute high altitudes and pH control during a high-intensity exercise, thus delaying fatigue. However, we cannot assume that these findings are cross-sectional under all environments since most studies only consider normoxic conditions or hypoxia induced by different devices.

The effects on anaerobic performance in multiple sprints and the influence of gender under HA environments have been poorly studied. The objectives of this study were to evaluate anaerobic performance in young women exposed to HA and to compare the physiological response to HA between women and men measured by ergospirometry.

## 2 Methods

### 2.1 Subjects

A total of 18 subjects, nine women and nine men (22.9 ± 3.2 years old), were enrolled in this pseudo-experimental, cross-sectional study. All volunteers were students of the University of Antofagasta, Chile. Eligibility criteria were as follows: the subjects must have a body mass index (BMI) between 18.5–24.9 kg/m2, no history of diagnosis of chronic or acute diseases, physically active (≥3 times/week of moderate and vigorous physical activity), and no history of mountain sickness and absence of absolute and relative contraindications for cardiopulmonary exercise testing (CPET) (American Thoracic Society and American College of Chest Physicians American College of Chest Physicians, 2003; Balady et al., 2010). Exclusion criteria were pregnancy and motor or musculoskeletal problems affecting the lower extremities. After detailed explanations by the investigators, all participants provided written informed consent. This study was carried out as part of the Network for Extreme Environment Research (NEXER), was approved by the Ethics Committee of the University of Antofagasta (ethical approval number: 050/2017), and was conducted in agreement with the Declaration of Helsinki.

### 2.2 Experimental procedure

Measurements took place under two different conditions, separated by two consecutive days, 1) at SL in Antofagasta, Chile, and 2) during the second full day of permanence at HA at 3.264 m.a.s.l. (barometric pressure of 692 mBar) in Caspana, Chile. Acute exposure was defined as the first 24 h from arrival to altitude (Pollock et al., 1993; Testing, 2015; Theunissen et al., 2022) in our study. During the first day of arriving at HA, the participants were instructed about the measurement procedures: investigators showed the Monark cycle ergometer in detail, accessories of the portable high-resolution spiroergometry system (MetaLyzer^®^ 3B-R2, CORTEX, Germany) such as a mask, spirometry sensor, gas analyzer, flow and volume sensor, and chest strap with a heart rate monitor (H10, POLAR, Finland). Instructions were also given before the test: wear comfortable and light clothing, with suitable shoes for exercising, comply with a 2-h fast, avoid active or passive smoking and alcoholic beverages for at least 4 h before the test, and do not perform vigorous exercise 1 day before the procedure (American Thoracic Society and American College of Chest Physicians American College of Chest Physicians, 2003; Balady et al., 2010). At night, they all slept at 3.264 m.a.s.l.

On the following day, prior to the test, the portable high-resolution spiroergometric system with breath-by-breath technology (MetaLyzer^®^ 3B-R2, CORTEX, Germany) was calibrated according to the barometric pressure measured with a barometer for adjustments according to the environmental conditions. Second, the flow and volume meters were calibrated with a 3L precision syringe and the oxygen (O_2_) and carbon dioxide (CO_2_) analyzers in tanks with high-precision gas mixtures, at least at two points (0% and 8% for CO_2_; 13% and 21% for O_2_) (American Thoracic Society and American College of Chest Physicians American College of Chest Physicians, 2003; Balady et al., 2010). The ergospirometer was also configured with the following predicted values: 1) maximum oxygen uptake according to the Wasserman weight algorithm; 2) maximum relative oxygen uptake based on the predicted maximum oxygen uptake value; 3) maximum oxygen pulse according to the Wasserman equation; 4) maximum heart rate according to the traditional formula for the bicycle test; 5) maximum minute ventilation according to individual predicted values, based on forced expired volume in 1 s (FEV 1), the previous spirometry result; 6) maximum respiratory rate according to Pollock et al. (1993) and the maximal work rate based on predicted maximal oxygen uptake; and 7) sets of predicted spirometry values based on the European Coal and Steel Community (ECSC). The multiple-sprint protocol was configured in MetaSoft^®^ Studio software of the equipment before the test. The anthropometric background of each subject and the barometric pressure of the place were entered.

**FIGURE 1:**
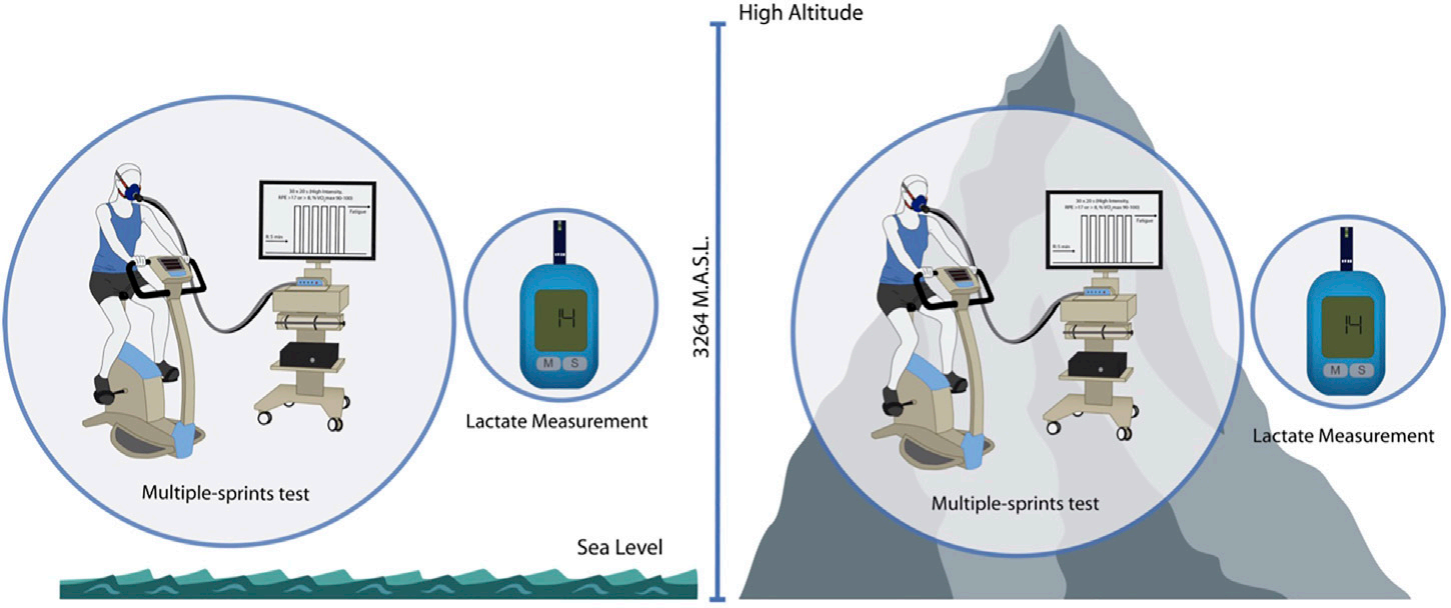
Experimental protocol overview. Schematic description of the experimental protocol. Measures at SL and HA were performed with 2 days of differences.

The participants’ body composition (weight, height, and body mass index), vital signs, and hemodynamic stability were assessed and entered into the personal file. All the participants underwent a multiple-sprint test under an identical setting, using the same devices and instruments, the same ambient temperature, and illumination, according to the reference procedure (Testing, 2015). After allowing a resting phase of at least 5 min sitting on the cycle ergometer, the participant started the test procedure, and the same operator conducted the test under strict monitoring and annotations of results. After the test, a 5-min active recovery sitting in the device was performed. Figure 1 shows the overview of the experimental protocol.

To guarantee the safety of the participants, mouthpieces with filters, disposable nose clips, and measures to control cross-infection of the professionals, such as access to hand washing and gel-alcohol, were used. To minimize the risk of adverse events, the protocol was conducted under the direct supervision of a general practitioner from the group of investigators with continuous monitoring of vital signs.

### 2.3 Multiple-sprint protocol performance

The multiple-sprint protocol consisted of 30 s of full work followed by 20 s of rest until fatigue in the cycle ergometer. In each sprint, revolutions per minute (rpm) were measured three times and recorded in the personal file.

Oxygen uptake (VO_2_), carbon dioxide production (VCO_2_), ventilation (VE), maximal ventilation (VE_max_), respiratory frequency (RR), respiratory exchange ratio VCO_2_/VO_2_ (RER), and minute ventilation to carbon dioxide output (VE/VCO_2_) were measured (MetaLyzer^®^ 3B-R2, CORTEX, Germany). The heart rate (HR) was monitored *via* a chest strap with a heart rate monitor (H10, POLAR, Finland), and arterial oxygen saturation was monitored using a pulse oximeter (Vantage 9590, Nonin, United States). Fatigue was defined and measured using the perceived exertion scale (RPE) of fatigue muscles of lower limbs by Borg scales (0–10) at the end of each sprint: RPE_mean_ was defined as the average of RPE achieved throughout the test until exhaustion, and RPE_max_ was defined as the maximal RPE achieved. Mean cadence (cadence_mean_) was defined as the average rpm achieved throughout the test until exhaustion, and maximal cadence (cadence_max_) was defined as the maximal rpm achieved. In addition, blood lactate was measured (lactate analyzer h/p cosmos model Scout–Sirius) at baseline, and the end of the test was registered on the personal file.

All the subjects had previously familiarized themselves with the test and knew the procedure. Two trained physicians supervised and conducted the test at sea level and high altitudes, monitored vital signs during all tests, monitored the appearance of reasons to interrupt the test (Balady et al., 2010), and applied the test completion criteria according to the subject’s perceived subjective effort (American Thoracic Society and American College of Chest Physicians American College of Chest Physicians, 2003).

### 2.4 Statistical analysis

The sample size was calculated using G*Power 3.1 software and considering the Wilcoxon test for two related samples to analyze the ventilatory response changes in women at SL and HA. Based on the VO_2max_ values at SL and HA reported for men in the study by Wehrlin and Hallén (2006), we assumed an effect size of d = 2.69. A significance level *α* = 0.05 and a power of 0.95 resulted in a minimum sample size per group of six. We decided to increase the sample size to nine per group to be able to consider other variables that could have a smaller effect size.

All statistical analyses were performed using MATLAB (MATLAB_R2022a, 2022). The significance level was set at *α* = 0.05 (two-sided) for all testing. Data are reported as marginal means and standard errors (mm ± SE).

The Shapiro–Wilk test was used to assess the data distribution; most of the data followed a normal distribution. For normally distributed data, the differences between women and men were evaluated by the Student’s *t*-test for two independent samples. For the non-normally distributed variables, the non-parametric Mann–Whitney U test for two independent samples was used. The differences between SL and HA were assessed by the Student’s *t*-test for two related samples for normally distributed variables and the Wilcoxon test for two related samples for non-normally distributed data. The effect size of the difference between groups and different conditions was computed as Cohen’s-d by Ruggero G. Bettinardi (mathworks, 2017). The relationship between variables was calculated using univariate linear regression. Finally, 1D data interpolation was used to estimate the VE and RER at a 1-s sampling for every subject between 4 and 100 s from the beginning of the test. The results were averaged at each sampling time, and the differences between groups were assessed using a Student`s *t*-test.

## 3 Results

A total of 18 enrolled participants, 9 women and 9 men, underwent a multiple-sprint test at both HA and SL. None of them had adverse events or reasons to interrupt the tests, and everyone successfully met the test completion criteria. Table 1 shows the sample’s descriptive data. Women and men differ significantly in weight and height (*p* < 0.005) but not in BMI, age, or VO_2peak_ (ml/kg/min^−1^) and are classified as good for both groups (Morrow et al., 2011; Rapp et al., 2018).

**TABLE 1:** Demographic characteristics include: age, weight, height, body mass index and VO2 peak.

Table 1. Sample’s demographic characteristic	Women	Men	*p*-value
Anthropometry			
Age (years)	23.4 ± 4.0	22.3 ± 2.1	0.781
Weight (kg)	56.1 ± 7.4	72.8 ± 13.4	0.005
Height (cm)	157 ± 0.1	170 ± 0.1	0.000
Body mass index (kg⋅m^−2^)	23.6 ± 2.6	24.9 ± 3.2	0.123
VO_2peak_ (ml/kg/min^−1^)	50.3 ± 9.6	55.5 ± 7.5	0.221

Descriptive data are reported as mean ± standard deviation. The differences between women (n = 9) and men (n = 9) groups were assessed by the Student’s *t*-test for two independent samples.

**FIGURE 2:**
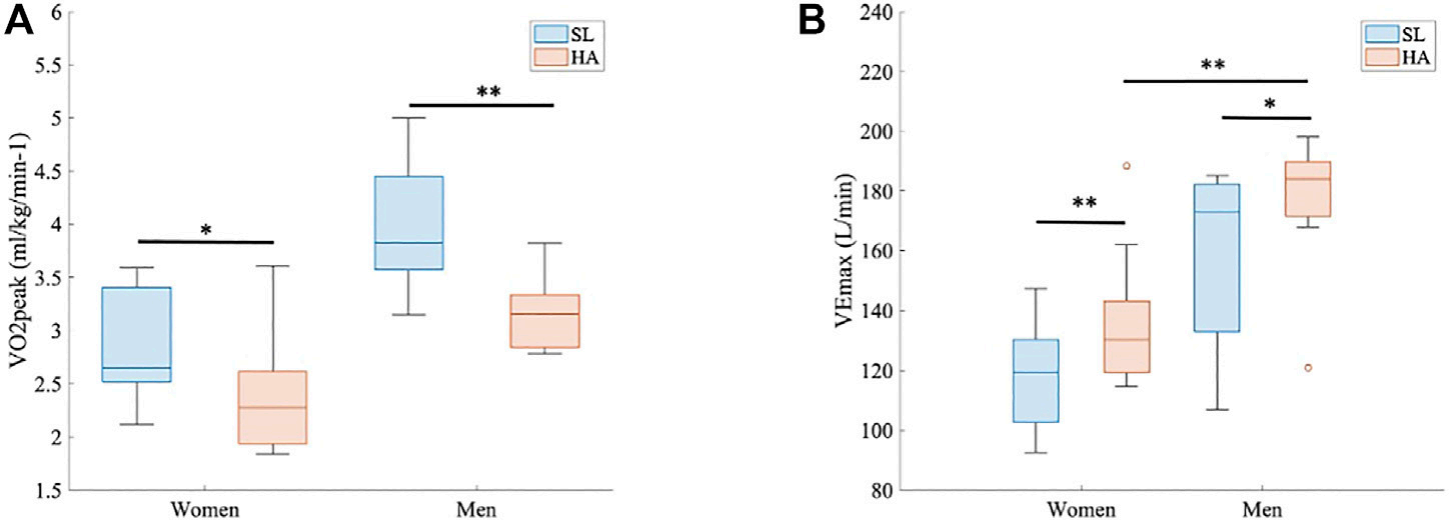
Multiple-sprint test results. **(A)** Peak oxygen uptake (VO_2peak_ (ml/kg/min^−1^) for both groups at both altitudes (*p* < 0.05 and d = 0.88 in women and *p* < 0.005 and d = 1.30 in men). **(B)** Maximal ventilation (VE_max_ (L/min) for both groups at both altitudes (*p* < 0.005 and d = 1.28 in women and *p* < 0.05 and d = 0.76 in men). Comparison of VE_max_ (L/min) between women and men at HA, 3.264 m.a.s.l (*p* < 0.005 and d = 1.68).

Figure 2A shows that multiple-sprint test results differ significantly by altitude. VO_2peak_ at the last sprint step decreased with the arrival at high altitudes in women (SL 50.25 ± 9.6 ml/kg/min^−1^ vs. HA 42.10 ± 5.9 ml/kg/min^−1^; *p* < 0.05, d = 0.88) and men (SL 55.46 ± 7.5 ml/kg/min^−1^ vs. HA 45.59 ± 9.5 ml/kg/min^−1^; *p* < 0.005, d = 1.30). The anaerobic performance considering realization by sprints, cadence_mean_, and cadence_max_ was maintained in both women and men. VE_max_ (L/min) also differed significantly by altitude (*p* < 0.005 and d = 1.28 in women and *p* < 0.05 and d = 0.76 in men). Also, VE/VCO_2_ increased significantly in both groups during exercise at HA (*p* < 0.005).

**FIGURE 3:**
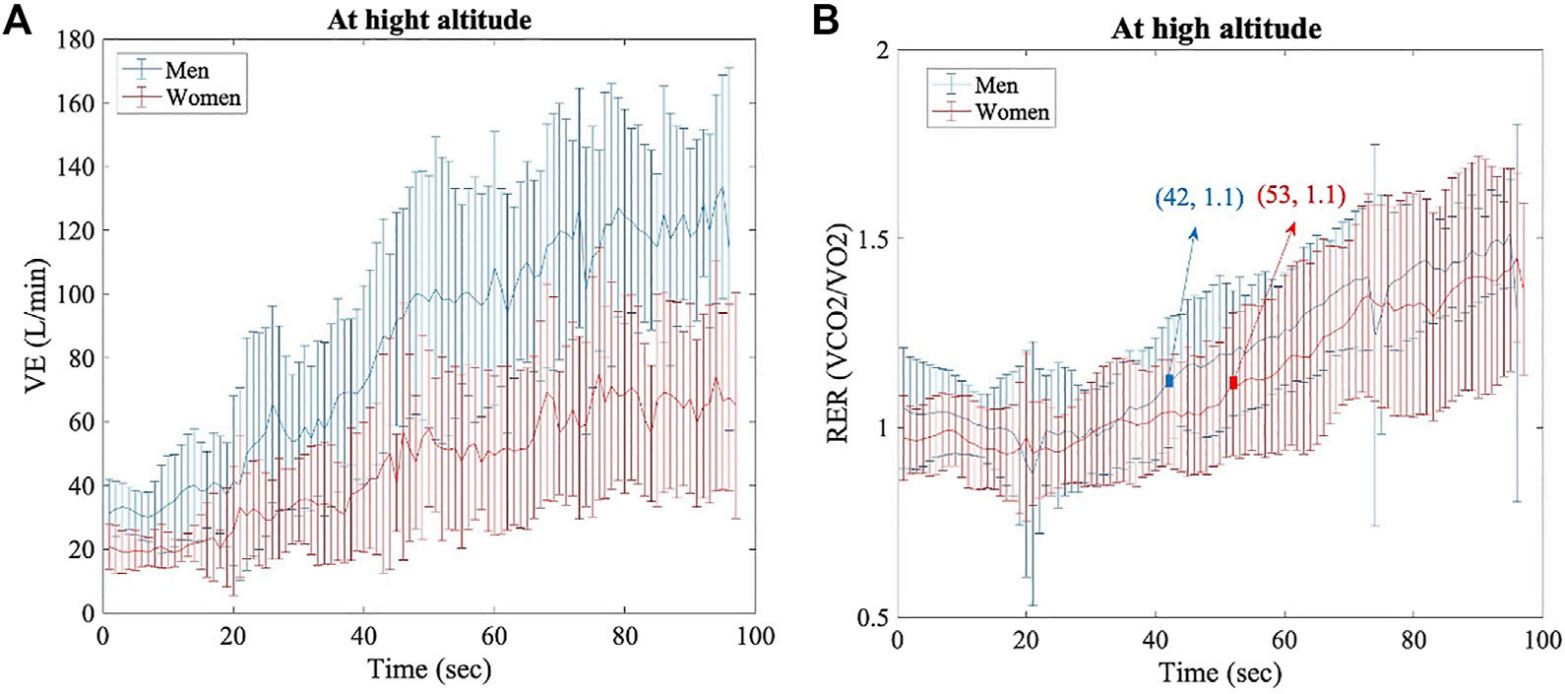
Per each timepoint of analysis (1 seg, range > 4seg, and < 100seg), for both groups (women and men) at HA. **(A)** Ventilation data, *p* < 0.05 for all time points. **(B)** Respiratory exchange ratio data, no significant differences.

**FIGURE 4:**
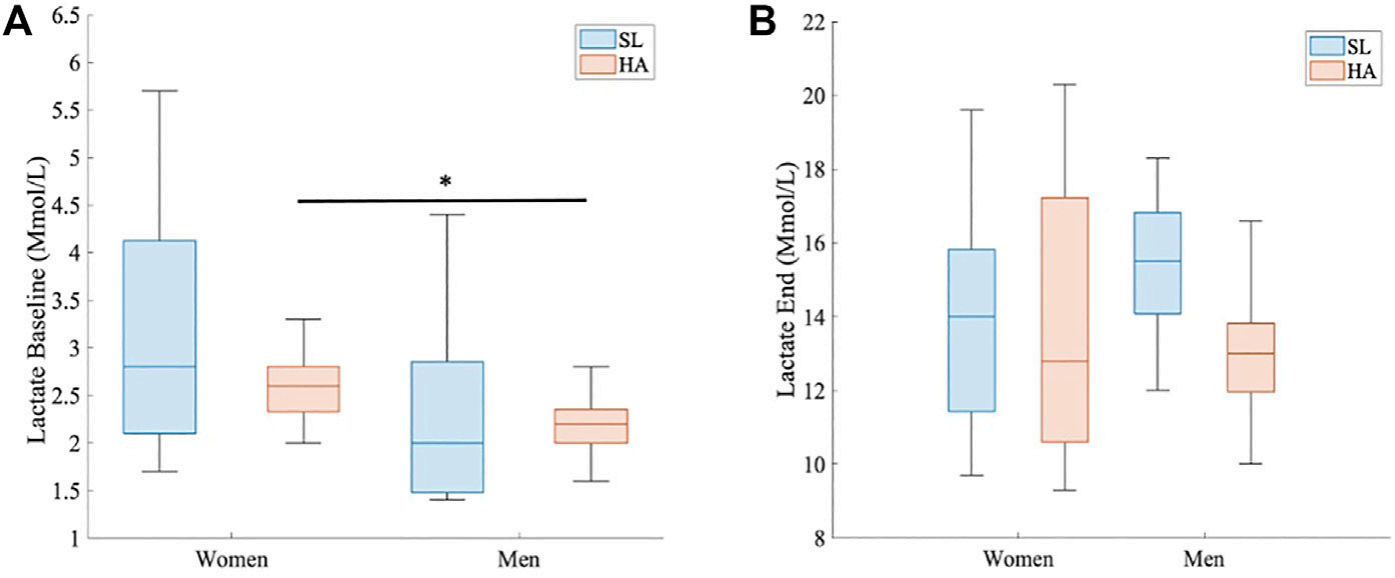
Comparison of lactate levels in HA. **(A)** Baseline lactate (Mmol/L) between women and men at HA, 3.264 m.a.s.l (*p* < 0.05 and d = 1.01). **(B)** End of test lactate (Mmol/L) between women and men at HA, 3.264 m.a.s.l.

At HA, the VE (L/min) statistically differed between women and men at every timepoint (1 s, range > 4 s and < 100 s; *p* < 0.05 for all points) (Figure 3A). VE_max_ was also different, as shown in Figure 2B (136.58 ± 23.9 L/min in women vs. 176.33 ± 23.3 L/min in men; *p* > 0.005, d = 1.68). RER did not show significant differences between groups under different conditions, but the point 1.1 was reached at 42 s by men and at 53 s by women (Figure 3B); RER > 1.1 has been accepted as a parameter of maximal exercise (Balady et al., 2010). The lactate baseline was significantly higher in women than in men after 24 h of permanence at HA (2.57 ± 0.4 Mmol/L, 2.18 ± 0.3 Mmol/L, respectively; *p* < 0.05, d = 1.01), but it did not differ at SL, where it showed much higher variability (Figure 4). There were no significant differences between women and men at SL and at HA in RPE_mean_ and RPE_max_ associated with lower extremity muscle fatigue during the anaerobic test to fatigue (*p* > 0.05).

**FIGURE 5:**
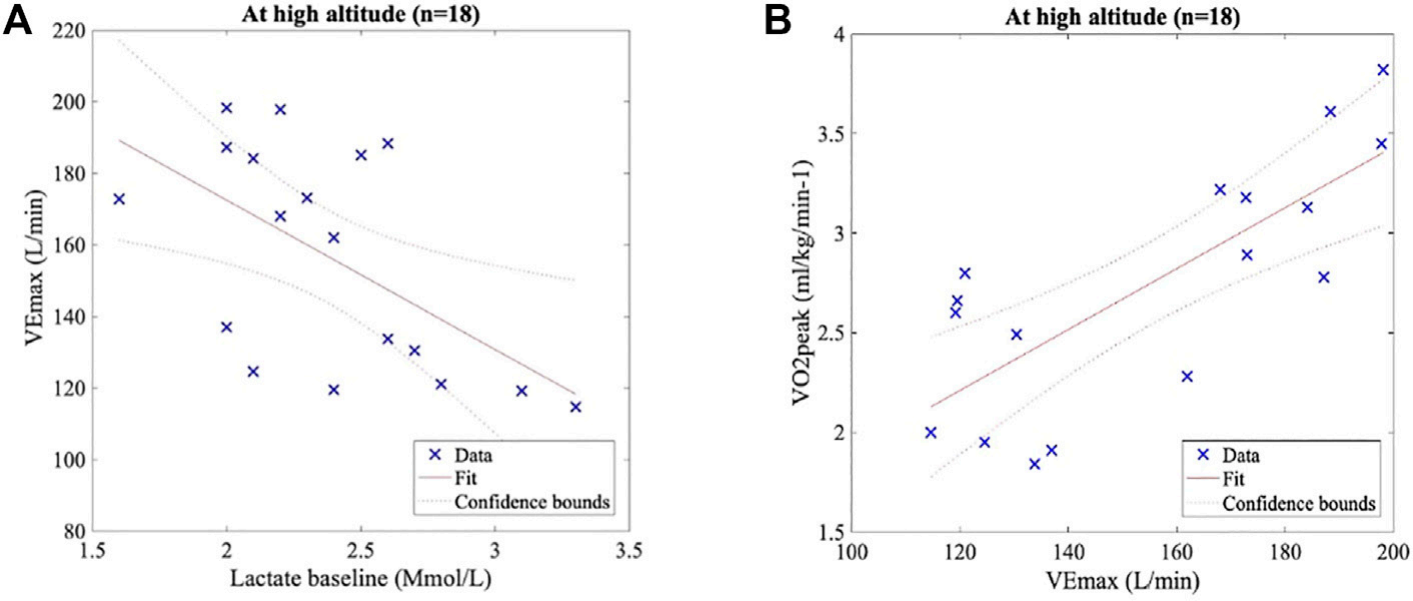
Pearson’s correlation test. **(A)** Lactate baseline and VE_max_ at HA present a significant inverse correlation (R^2^ = 0.33, slope = −41.7, and *p* < 0.05). **(B)** VEmax and VO_2peak_ at HA show a significant positive correlation (R^2^ = 0.60, slope = 0.02, and *p* < 0.001).

A linear regression model fitted between baseline lactate and VE_max_ at HA showed a significant inverse correlation (R^2^ = 0.33, slope = -41.7; *p* < 0.05). A strong linear positive correlation was observed between VE_peak_ and VO_2peak_ (R^2^ = 0.60, slope = 0.02; *p* < 0.001), and both results are shown in Figure 5.

## 4 Discussion

The main findings of this study are that the women have higher lactate levels in the first 24 h of exposure to high altitudes than men, followed by a lower VR during and at the end of a multiple-sprint test. It is important to point out how these findings are only manifested at HA since no significant differences between men and women were found at SL or normoxia.

It is likely that extreme environmental conditions, such as high altitudes, added to a repetitive maximal effort, such as a multiple-sprint test, reveal underlying physiological aspects of the cardiorespiratory response and human physical capacity. The VO_peak_ behavior in this study during an acute exposure to geographic altitude does not differ from studies carried out under similar conditions (Buskirk et al., 1967; Beall, 2007; Grocott et al., 2009). The lower PiO_2_ leads to a drop in the arterial oxygen content (CaO_2_) and its availability in the tissues, which causes a decrease in VO_2peak_ compared to SL.

According to our findings, repetitive maximal efforts, such as a multiple-sprint test, are associated with an increased ventilatory response and termination of the test in women exposed to HA, which could be attributed to a rapid fatigue of the respiratory musculature. Although no significant differences in anaerobic maximal performance were observed between genders, the data collected show significant differences in VR (VE_max_ and VE). Our findings are consistent with those reported by Goldberg et al. (2017) from a sample of 551 healthy university students (61.3% women) that measured the VR using a re-breathing system simulating hypoxia through masks and a closed circuit until reaching an arterial oxygen saturation (SpO_2_) of 75%. They observed that women have an approximately 50% lower VR than men (*p* < 0.001) and lower baseline minute ventilation. After controlling the body surface area (BSA), the difference decreases but remains higher for men (31.2%). This allows us to confirm that VR and gender are strongly correlated besides the weight and height differences among our participants. As for exercise, it has been observed that most of the aerobically trained women (VO_2max_ > 50 ml/kg/min^−1^) and sedentary women develop exercise-induced arterial hypoxemia, defined as decreases in PaO_2_ higher than 10 mmHg during exercise (constant load test, ∼85% VO_2max_) than that at rest (Dominelli et al., 2013). An experiment with heliox breathing in the same study demonstrated the mechanical ventilatory constraints of women as smaller lung volumes and airway diameters cause a poor reserve that increases ventilation effort, which may result in increased susceptibility to hypoxemia during exercise.

Some studies postulate the importance of neural or central control in VR during exercise (Harms, 2007; Moreno et al., 2014; Luks, 2015; Holwerda and Vianna, 2019; Lam et al., 2019). The metaboreflex, i.e., the possibility that the respiratory center is modified by afferent inputs from metabolic feedback originating in the active skeletal muscle, has received increasing attention during the last century (Holwerda and Vianna, 2019; Lam et al., 2019). Dempsey et al. (2006) hypothesized that high energy (intensities greater than 80% of VO_2max_) and circulatory demand by sustained hyperventilation are necessary to activate the metaboreflex, provoking an increase in the sympathetic vasoconstrictor outflow, causing a reduced blood flow to skeletal muscles. They postulate that during high-intensity exercise, a competition between two large muscle groups is generated to satisfy their oxygen demands: on one hand, the diaphragm, and on the other, the active locomotor muscles. In our study, repetitive high-intensity exercise is performed together with sustained hyperventilation due to arrival at HA, so women may have greater metaboreflex activation than men to maintain a normal pH and overcome hypoxia. The 24 h elapsed from arrival at HA with a greater ventilatory effort triggers the recruitment of the respiratory tract’s accessory muscles, which has been shown to ultimately lead to inefficiency by increasing the oxygen cost of breathing (Ward et al., 1988; Dempsey et al., 2006).

Another point to consider is the energy demand of the exercise and how it is satisfied under this environmental condition. Semenza et al. (1996) studied the expression of genes and glycolytic enzymes in non-hypoxic cells (for 24 h to 20% O_2_) and hypoxic cells (for 48 h to 1% O_2_) and showed that during exposure to hypoxia, the cells use glycolysis as an energy substrate for the expression of HIF-1. We know that aerobic glycolysis is a fairly efficient oxidative mechanism for the production of ATP. However, under hypoxic conditions, the preferred mechanism is likely anaerobic glycolysis, which generates an accumulation of hydrogen ions and lactate, especially during our first 24 h of arrival to HA. In our study, the anaerobic metabolism can be appreciated indirectly through the measurements of blood lactate and RER. The latter reached values over 1.1 earlier in men, showing a higher tolerance to anaerobic glycolysis for a longer time and indicating a better capacity for hydrogen ion buffering at high-intensity exercises. We do not know studies on gender differences in the expression of HIF-1, but according to our findings, it can be hypothesized that women may have this metabolic expression earlier than men, causing this increase in baseline lactate levels at HA, which we observed to be inversely correlated with VE during the anaerobic test. Another physiological mechanism could derive from the differences in muscle mass between women and men; the greater number of type IIa muscle fibers could facilitate in men a more gradual aerobic–anaerobic transition during the exposure to hypobaric hypoxia in men. However, this highly depends on the fitness performance and the type of sport or resistance training practiced by each subject (LeBrasseur et al., 2011).

On the other hand, ventilatory response differences can be explained by hormonal response during the menstrual cycle. Their influence on the central neural control of breathing, serotonergic system, and their release in the hypothalamus, have been related to changes in progesterone and estradiol levels, increasing serotonin in the hypothalamus and consequently changing the respiratory rate. Nevertheless, these hormones alone cannot generate ventilatory changes, but it has been seen that they can influence when they are found simultaneously, acting as a respiratory stimulant (Behan et al., 2003; Dominelli et al., 2013). On the contrary, a recent study with 10 healthy young females (age 23 ± 5 years) measured estrogen and progesterone daily and chemosensitivity variability across one complete cycle. Using the hypoxic ventilatory response by using a face mask, 100% molecular nitrogen was gradually titrated, lowering the hypoxic gas (FiO_2_ of 15%) until SpO_2_ reached 80% at rest and submaximal exercise (40% of maximum power). At rest, the ventilatory response was unaffected by the menstrual cycle phase and was not different between men and women. Moreover, the sensitivity to chemical stimuli was unaffected. In addition, similar responses were found during the cycle ergometer exercise test. Ventilation corrected for both the body surface area (BSA) and power outputs showed no differences, probably because the magnitude of the hormone changes is insufficient to modify the chemosensitivity (Macnutt et al., 2012).

**FIGURE 6:**
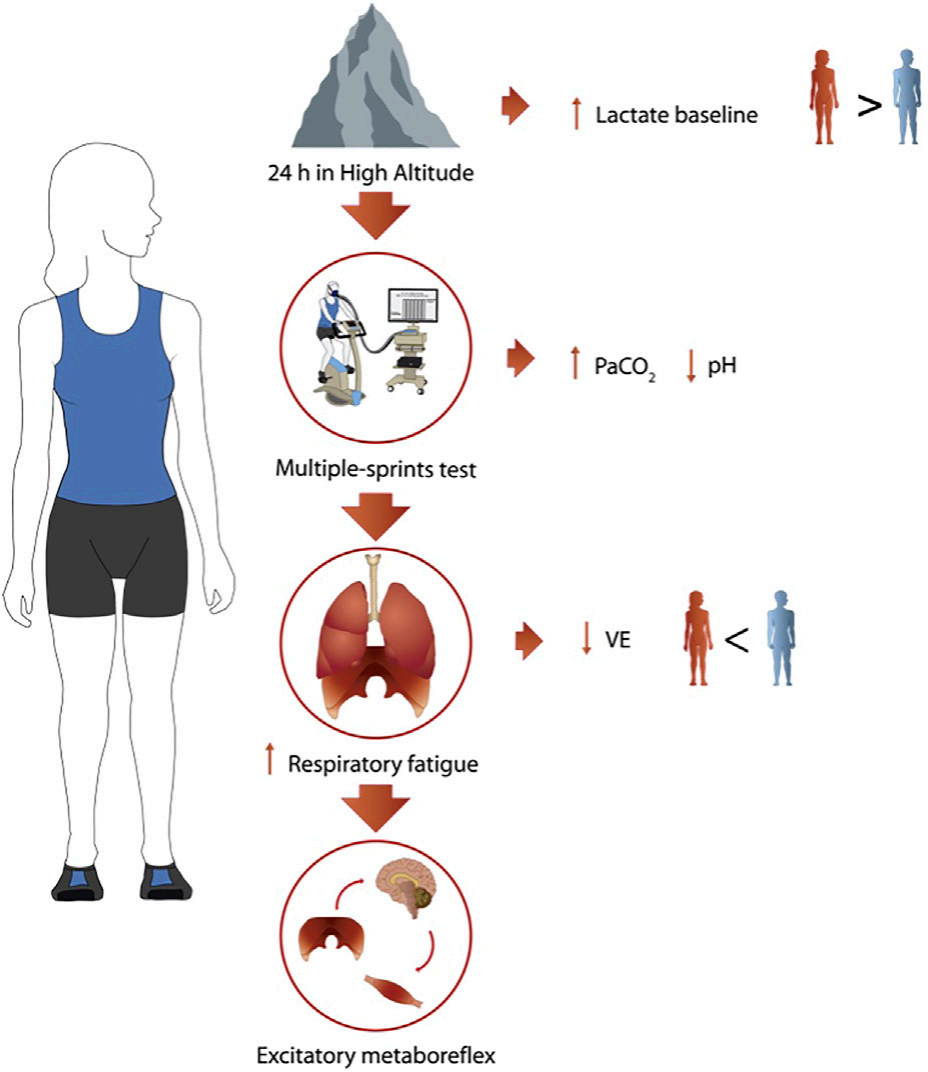
Summary of the main results. Schematic description of main findings at HA in women (*n* = 9).

The present study provides insights into the mechanisms behind the reduced ventilatory response observed in women during an anaerobic exercise test at HA. Hypoxia conditions showed an exacerbated ventilatory drive response related to a greater work of breathing, which may suggest the presence of a neural, locomotor, and respiratory connection, which is possibly less efficient in women, as shown in Figure 6. All the mentioned comparisons should be taken with caution because the simulated hypoxic conditions differ from hypobaric hypoxia, so studies of the physiological behavior of women under extreme environments should be encouraged.

This study has the limitation to the small sample, associated with the greater logistic of this project and lack of randomization. Another point to consider is the lack of continuous measurement of blood lactate levels, as well as arterial blood gases (pH, PaO_2_, and carbon dioxide blood pressure, PaCO_2_). Additionally, no inquiries were made about the women’s menstrual cycle or contraceptive use. Acute mountain sickness was not measured with a quantitative instrument; however, headache, gastrointestinal symptoms, and vertigo/dizziness were assessed through an interview upon arrival to HA, after the first night and before the test.

The main strength of this study was the execution of an anaerobic multiple-sprint protocol with 20 s of intermittent recovery with ergospirometry according to gender since very few studies have been carried out under real conditions of geographic altitude, which incorporate this type of exercise, technology applied, and female participants.

## 5 Conclusion

Acute HA exposure alters anaerobic performance during a multiple-sprint test in healthy young women. We have shown that during the anaerobic multiple-sprint protocol, there is no recovery after 20 s, where the ventilatory response and lactate are protagonists of the performance and gender differences. Women showed higher lactate levels and lower lung ventilation in the test, possibly associated with differences in the fatigue-induced metaboreflex of the respiratory muscles and early aerobic–anaerobic transition during acute exposure to hypobaric hypoxia in women. These results on multiple-sprint performance and the influences of gender under hypoxic environments deserve further investigation.

## Data Availability

The raw data supporting the conclusions of this article will be made available by the authors, without undue reservation.
